# How to get doctors to hand hygiene: nudge nudge

**DOI:** 10.1186/2047-2994-4-S1-O51

**Published:** 2015-06-16

**Authors:** A Kwok, M-L McLaws

**Affiliations:** 1 School of Public Health and Community Medicine, UNSW Medicine, Sydney, Australia

## Introduction

The Nudge Theory has been widely used to improve healthy behavior [1,2] and works as an external cue to memory [3]. The Nudge Theory is based on behavioural economics models that has been applied to move communities towards rational targeted purchasing and ecological preferred behaviour patterns [1,2]. Consumers have been nudged successfully towards lower electricity purchasing patterns through displays of their past and current purchasing pattern.

## Objectives

To engage medical staff towards improved hand hygiene compliance.

## Methods

An automated hand hygiene surveillance system was installed in an Australian tertiary teaching hospital. The clinicians were taught to access a dashboard for daily compliance rates to be discussed at hand-over meetings and to use a seven-step approach that included nudging each other on the wards with *“Doctor*, *take a moment*”. Feedback from clinicians about nudging and rates on both wards from June 2014 to February 2015 were compared.

## Results

During the run-in period prior to introducing nudging the baseline compliance rate was 18% on ward C and 37% on ward D. Preliminary results indicated one ward has improved by 32 percentage points while compliance on ward C remained stable at 15%. Clinicians on ward D reported that they were comfortable working as a team to nudge each other towards a goal of improved daily compliance and it was fun. Conversely, ward C clinicians reported a discomfort with nudging each other and were observed to have a different ward culture than ward D. We will discuss the difference in the staff and leadership on the wards that may explain the opposing attitudes towards nudging.

## Conclusion

Nudging daily compliance rates by nurses and doctors resulted in collegiality and developed a ‘team consciousness’ about improvement in compliance. Changing hand hygiene compliance must work with different organization cultures on wards for effective change management.

## Disclosure of interest

A. Kwok Conflict with: Deb Australia, Debgroup UK provided the automated system to collect daily compliance rates. The authors declared have no other conflict of interests. , M.L. McLaws Conflict with: Deb Australia, Debgroup UK provided the automated system to collect daily compliance rates. The authors declared have no other conflicts of interest.

## References

[B1] Thaler R Sunstein C Nudge: Improving decisions about health, wealth and happiness 2009 Penguin Books Ltd UK

[B2] Vallgarda S Nudge- A new and better way to improve health? Health Policy 2012 104 2 200 203 10.1016/j.healthpol.2011.10.013 22113151

[B3] Sax H Clark L Mental models: a basic concept for human factors design in infection prevention J. Hospital Infect 2015 89 4 335 339 10.1016/j.jhin.2014.12.008 25676111

